# Phenotypic Diversity of *Lactobacillus sakei* Strains

**DOI:** 10.3389/fmicb.2018.02003

**Published:** 2018-08-28

**Authors:** Chiara Montanari, Federica Barbieri, Michael Magnani, Luigi Grazia, Fausto Gardini, Giulia Tabanelli

**Affiliations:** ^1^Interdepartmental Center for Industrial Agri-Food Research, University of Bologna, Cesena, Italy; ^2^Department of Agricultural and Food Sciences, University of Bologna, Bologna, Italy

**Keywords:** *Lactobacillus sakei*, primary metabolism, amino acid metabolism, metabolic diversity, growth potential

## Abstract

*Lactobacillus sakei* is a lactic acid bacteria (LAB) species highly adapted to the meat environment. For this reason, selected strains are often used as starter culture in the production of fermented sausages, especially in Mediterranean countries. It often represents the dominant species in these products and can maintain its viability during all the ripening period, which can take also some months. This ability is guaranteed by the possibility of the species to obtain energy through pathways active even when hexoses are depleted. This species is characterized by a relevant genetic and phenotypic diversity and its metabolism can be further affected by the growth condition applied. In this work we investigate the metabolic responses of six different *L. sakei* in a synthetic medium (DM) containing defined amounts of amino acids in relation to temperature and NaCl concentration. In addition, the activities of cells pre-grown in presence of glucose o ribose were tested. Arginine was efficiently up-taken with the exception of the type strain DSMZ 20017^t^. Other amino acids (i.e., serine, asparagine, cysteine, and methionine) were metabolized through potentially energetic pathways which start from pyruvate accumulation, as demonstrated by the organic acid accumulation trend in the condition tested, especially in DM without sugar added. The presence of excesses of pyruvate deriving from amino acids lead to the accumulation of diacetyl and acetoin by all the strains when sugars were added. This approach allowed a deeper insight into the phenotypic variability of the species and improved the comprehension of the metabolic pathways adopted by *L. sakei* to survive and grow in restrictive conditions such as those found in fermented sausages during fermentations. Thus, the results obtained are useful information for improving and optimizing the use of such strains as starter culture for these products.

## Introduction

*Lactobacillus sakei* is a lactic acid bacteria (LAB) highly adapted to grow in meat environments in which it can outcompete undesired microorganisms, including pathogenic species (Chaillou et al., 2013). For this reason, it is often responsible for natural fermentation of dry fermented sausages (Hugas et al., 1993). Because of this aptitude, selected strains of this species are widely used as starter cultures in meat fermentation together with strains belonging to the species *L. curvatus*, *Pediococcus pentosaceus*, and *P. acidilactici* (Hammes and Hertel, 1998; Champomier-Vergès et al., 2001). The main energy sources are sugars: hexose fermentation is homolactic while pentoses(such as ribose) are fermented through the heterolactic pathway (McLeod et al., 2008; Rimaux et al., 2011b). Nevertheless, the ability of the species to dominate the microbiota of fermented sausages for several weeks, when the hexoses are depleted after few days from the production, underlines its ability to use other substrates to obtain energy for growth and survival (Cocconcelli and Fontana, 2010). Within the species, two subspecies are recognized (Torriani et al., 1996), *L. sakei* ssp. *sakei*, and *L. sakei* ssp. *carnosus*, which differ for the presence of specific soluble cell proteins. However, Chaillou et al. (2013) recently suggested that members of this species derived from three ancestral lineages determined by independent selection scenarios.

*Lactobacillus sakei* is characterized by a wide genetic and phenotypic diversity. In the last years, its genome has been sequenced and a high variability in the dimension has been observed. The mean genome size was 2,020 kb with a variation of about 25% (from 1,814 to 2,309 kb) (Chaillou et al., 2005, 2009).

The genome analysis evidenced some specific traits of this species, which can explain its high adaptation to meat environment (Claesson et al., 2007). Studies have been carried out on the ability to catabolize arginine (Rimaux et al., 2011a, 2012), the purine nucleoside metabolism (Rimaux et al., 2011b) and the high adaptability to some adverse environment conditions such as cold, oxidative, and high salt stresses (Duhutrel et al., 2010; Guilbaud et al., 2012; Belfiore et al., 2013). In particular, the utilization of the ribose present in nucleosides and the activation of the arginine deiminase (ADI) pathway can be additional energy sources giving a competitive advantage in matrices with low fermentable sugar concentration (McLeod et al., 2017).

Amino acids play a key role in explaining *L. sakei* survival and growth in meat. The species is auxotrophic for all amino acids except aspartic and glutamic acids, which can be obtained by the deamination of asparagine and glutamine, respectively (Chaillou et al., 2005). The absence of the metabolic pathways for amino acid synthesis and the absence of transaminases are a result of the adaptation of *L. sakei* to meat, a substrate extremely rich in these molecules, which can be up-taken as free amino acids or short peptides (Sinz and Schwab, 2012). In addition to their role in protein synthesis, amino acid can be involved in other extremely important pathways for the overall cell metabolism. They can in fact contribute to energy generation (Fernández and Zúñiga, 2006) as well as to the production in sausages of aroma compounds or undesired substances such as biogenic amines (Suzzi and Gardini, 2003; Smid and Kleerebezem, 2014).

The genomic variability of *L. sakei* has already been demonstrated. Nevertheless, as observed by Sinz et al. (2013), the metabolic activities of a microorganism (or of a species) can be only hardly deduced from the genome and additional biochemical efforts are needed to evaluate the phenotypic potential of strains of industrial interest.

In this work, the growth performances of six *L. sakei* strains in relation to temperature and salt concentration were modeled. Moreover, in order to understand how a primary energy source could affect the overall strain metabolism, the strains were inoculated in a defined medium (DM) containing 20 free amino acids and added or not with sugars (glucose or ribose). After 24 h of incubation, analyses were carried out in order to quantify the cell number, pH and the amounts of amino acids in the DM, as well as the organic acid produced (L-lactate, D-lactate, acetate, and formate). In addition, also C4 volatile compounds produced by the strains (diacetyl and acetoin) were evaluated.

## Materials and Methods

### Strains

Six *L. sakei* strains were employed in this work. The type strain DSMZ 20017^t^ (DSMZ, Braunschweig, Germany), the collection strain DSMZ 6333, isolated form vacuum-packaged pork meat, a strain (Chr82) provided by Chr. Hansen (Parma, Italy) and three strains belonging to collection of Dipartimento di Scienze e Tecnologie Agroalimentari (University of Bologna). In particular, BR3 and TA13 were isolated from spontaneous fermented pork sausages produced in Emilia-Romagna region while the strain CM3 were isolated from dried camel meat produced in Algeria (Gozzi et al., 2017).

The *L. sakei* strains were maintained in de Man Rogosa and Sharp (MRS) medium (Oxoid, Basingstoke, United Kingdom) with 20% (w/v) glycerol at −80°C until usage. Before the experiments, strains were pre-cultivated twice in MRS medium for 24 h at 30°C.

### Growth Modeling at Different Temperature and Salt Concentration

The *L. sakei* strains were inoculated in MRS (initial concentration ∼4 log cfu/ml) and their growth in relation to temperature and NaCl concentration was monitored through the variation of optical density at 600 nm (OD_600_), measured with a UV-VIS spectrophotometer, 6705 UV-Vis (Jenway, Stone, United Kingdom). Medium pH at the end of growth was also measured (pH meter Basic 20, Crison, Modena, Italy) and the acidification activity was expressed as pH decrease with respect to the initial value (about 6.5). In particular, the effect of temperature was monitored by incubating the cultures from 5 to 40°C (with a step of 5°C) while the effect of NaCl was determined at 30°C adding 0, 2, 4, 6, and 8% (w/v) of NaCl to MRS before sterilization.

The OD_600_ data were fitted with the Gompertz equation as modified by Zwietering et al. (1990).

$$y={Ae}^{-e^{\left[\left(\frac{\mu_{\max}e}{A}\right)(\lambda -t)+1\right]}}$$

where *y* is the OD_600_ at time *t*, *A* represents the maximum OD_600_ value reached, μ_max_ is the maximum OD_600_ increase rate in exponential phase and λ is the lag time.

### Inoculum in Defined Medium (DM)

**FIGURE 1:**
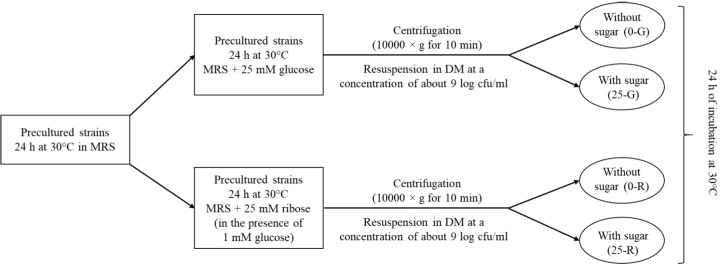
Experimental plan for defined medium (DM) trial.

After a pre-growth in MRS medium at 30°C for 24 h, the *L. sakei* strains were cultured in a modified MRS containing 25 mM of glucose or 25 mM of ribose as fermentable carbohydrates, for 24 h at 30°C. In the case of MRS added with ribose, according to the observation of McLeod et al. (2008), a small amount of glucose (1 mM) was also used. After growth, the cells were collected in the early stationary phase by centrifugation (10,000 × *g* for 10 min), washed twice with physiological solution (0.9%, w/v NaCl) and re-suspended at a concentration of about 9 log cfu/ml in different defined media (DM), sterilized by filtration. The composition of DM (adapted from Lauret et al., 1996), containing defined amounts of amino acids (added at a concentration of 0.2 g/L), vitamins and growth factors, is reported in **Table 1**. Cells pre-grown in the presence of glucose were re-suspended in DM containing glucose 25 mM (25-G) or without sugar (0-G). Similarly, cells pre-grown in the presence of ribose were re-suspended in DM containing ribose 25 mM (25-R) or without sugar (0-R) (**Figure 1**). Samples were incubated at 30°C for 24 h. Three independent samples for each conditions were analyzed.

**Table 1:** Defined medium (DM) composition.

Components	Concentration	Components	Concentration
**Macro components**	**mM**	**Amino acids**	**mM**
KCl	10.06	Glutamic acid (glu)	1.36
MnSO_4_	0.05	Aspartic acid (asp)	1.50
MgSO_4_	1.66	Alanine (ala)	2.25
Na_2_HPO_4_	12.33	Arginine (arg)	1.15
Tween 80	1.65	Asparagine (asg)	1.51
		Cysteine (cys)	1.65
Vitamins	μM	Glutamine (glm)	1.37
Thiamine HCl	3.0	Glycine (gly)	2.66
Folic acid	0.5	Histidine (his)	1.29
Riboflavin	2.7	Isoleucine (ile)	1.53
Calcium pantothenate	4.6	Leucine (leu)	1.53
Nicotinic acid	8.1	Lysine (lys)	1.37
Pyridoxal	3.0	Methionine (met)	1.34
P-amino benzoic acid	2.9	Phenylalanine (phe)	1.21
		Proline (pro)	1.74
Nucleotides	mM	Serine (ser)	1.90
Adenine	0.037	Threonine (thr)	1.68
Guanine	0.046	Tryptophan (try)	0.98
Uracil	0.089	Tyrosine (tyr)	1.10
		Valine (val)	1.71

*The concentration of the components was adapted from Lauret et al. (1996).*

#### Survival Analysis and DM pH

DM samples were incubated at 30°C and, after 24 h, cell survival was assessed by plate counting in MRS agar (Oxoid) incubated for 48 h at 30°C. In addition, pH was monitored using a pH meter Basic 20 (Crison).

#### Diacetyl, Acetoin, and Ethanol Determination

Volatile organic compounds of samples were monitored, after 24 h of incubation at 30°C in 10 mL sterilized vials sealed with PTFE/silicon septa, using a gas-chromatography-mass spectrometry coupled with solid phase microextraction (SPME-GC-MS). An Agilent Hewlett-Packard 6,890 GC gas-chromatograph, equipped with a MS detector 5970 MSD (Hewlett-Packard, Geneva, Switzerland) and a CP-WAX 52CB 50 m × 0.32 mm × 1.2 μm fused silica capillary column was used (Agilent Technologies, Santa Clara, CA, United States). The samples were pre-equilibrated for 10 min at 45°C, after that a fused silica fiber covered by 50/30 μm divinylbenzene/carboxen/polydimethylsiloxane (DVB/CAR/PDMS, StableFlex) (Supelco, Steinheim, Germany) was introduced into the headspace for 40 min. Adsorbed molecules were desorbed in the gas-chromatograph for 10 min. The conditions were the same reported by Montanari et al. (2016). Blanks (empty vials) were injected regularly to monitor possible carry over. An auto-tune of the GC-MS was daily carried out prior to the analysis to ensure optimal GC-MS performance. The volatile compounds were identified by computer matching of mass spectral data with those of compounds contained in NIST 2011 mass spectral library (Scientific Instrument Services, Ringoes, NJ, United States). Moreover, for the most important compounds, the mass spectrum identification was confirmed by injection of the pure standards (Sigma-Aldrich, St. Louis, MO, United States) in the same conditions. The compounds were reported as ratio between each molecule peak area and peak area of the internal standard, 4-methyl-2-pentanol (Sigma-Aldrich, St. Louis, MO, United States), added at a final concentration of 33 mg/kg.

#### Organic Acids and Biogenic Amine Production

The concentration of organic acids in the samples was determined by an HPLC (PU-2089 Intelligent HPLC quaternary pump, UV-VIS multiwavelength detector UV 2070 Plus; Jasco Corp., Tokyo, Japan) and a manual Rheodyne injector with a 20 μL loop (Rheodyne, Rohnert Park, CA, United States), equipped with a Bio-Rad Aminex (Bio-Rad Laboratories, Hertfordshire, United Kingdom) HPX-87H column (300 mm × 7.8 mm). The analysis was performed in isocratic conditions, using mobile phase H_2_SO_4_ 0.005 M, with rate flow of 0.6 ml/min and temperature of 65°C. The UV detector was set at 210 nm. Chromatographic peaks were identified by comparing retention times with those of standards (Sigma-Aldrich, St. Louis, MO, United States) and quantification was carried out by using the external standard method.

Biogenic amines were detected and quantified according to the HPLC method reported by Bargossi et al. (2015). Under these analytical conditions, the biogenic amines detected were 2-phenylethylamine, putrescine, cadaverine, histamine, tyramine, spermine, and spermidine.

#### Amino Acids Quantification

For the variation of amino acid concentrations in DM media, samples were subjected to an AccQ-Fluor Reagent (AQC, 6-aminoquinolyl-N-hydroxysuccinimide carbamate) derivatization (Waters Corp., Milford, MA, United States) according to the manufacturer’s protocol. AQC was reconstituted at a final concentration of 10 mM in acetonitrile, included in the AccQ-Fluor Reagent Kit (Waters Corp.). Briefly, 10 μL of samples were derivatizated with 70 μL of AccQ-Fluor Borate Buffer (Waters Corp.) and 20 μL of reconstituted reagent. The samples were heated to 55°C for 10 min. The amino acids content was analyzed using an HPLC (PU-1580 Intelligent HPLC pump, Intelligent Fluorescence Detector FP-1520 and Intelligent Sampler AS-2055 Plus, with 10 μl loop; Jasco Corp.). Separation of amino acids was obtained using AccQ-Tag^TM^ column (3.9 mm × 150 mm) for amino acid analysis (Waters Corp.). A gradient elution was performed maintaining a column temperature of 30°C and using two mobile phases: A (100 ml of AccQ-Tag Eluent A concentrate (Waters Corp.), diluted 1:10 with H_2_O for chromatography (Sigma-Aldrich, St. Louis, MO, United States) and B (60% acetonitrile and 40% H_2_O for chromatography) (Sigma-Aldrich, St. Louis, MO, United States) with a flow rate of 1 ml/min. The fluorescence detector was set at excitation wavelength of 250 nm and emission wavelength of 395 nm.

### Statistical Analysis

The estimates of parameters of Gompertz equation were obtained using the non-linear regression procedure of Statistica for Windows (Statistica 8 software, 2006; StatSoft, Tulsa, OK, United States). ANOVA was performed using the one-way procedure of Statistica and significant differences between the conditions were evaluated with the LDS test (*p* ≤ 0.05). The heat map was obtained in the statistical environment R (R Development Core Team, Vienna, Austria).

## Results and Discussion

### Growth Modeling at Different Temperature and Salt Concentration

**FIGURE 2:**
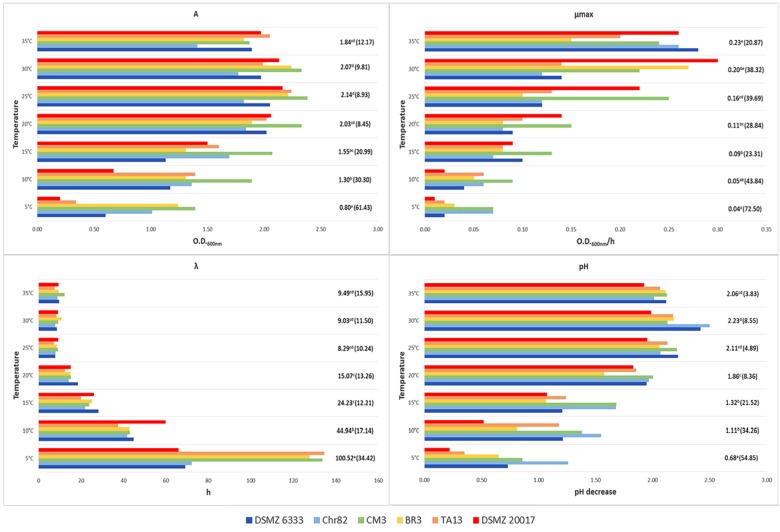
Growth of *L. sakei* strains in MRS at different temperatures: estimates of the Gompertz parameters and pH decrease (initial value about 6.0). *A* represents the maximum OD_600_ value reached, μ_max_ is the maximum OD_600_ increase rate in exponential phase and λ is the lag time. For each temperature, the mean value is reported and the relative coefficient of variation is indicated between brackets. Means with the same letter are not statistically different (*P* > 0.05) according to the *post hoc* (LSD test) comparison of ANOVA.

**FIGURE 3:**
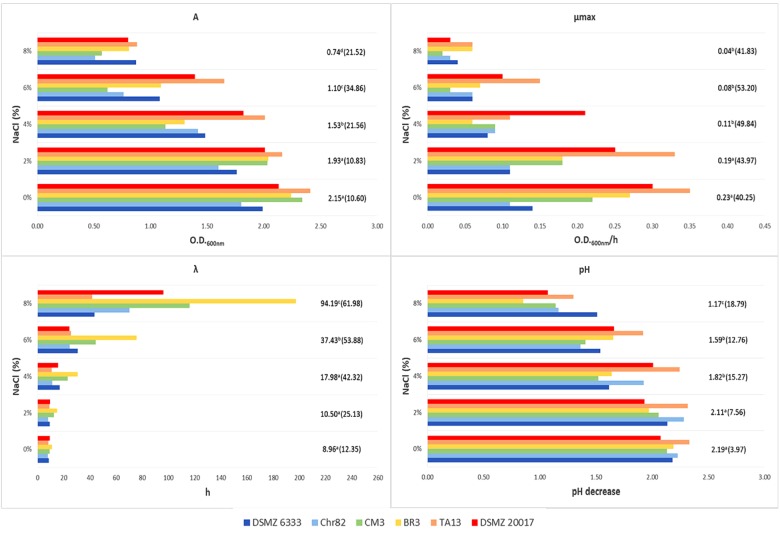
Growth of *L. sakei* strains in MRS at different salt concentrations: estimates of the Gompertz parameters and pH decrease (initial value about 6.0). *A* represents the maximum OD_600_ value reached, μ_max_ is the maximum OD_600_ increase rate in exponential phase and λ is the lag time. For each NaCl concentration, the mean value is reported and the relative coefficient of variation is indicated between brackets. Means with the same letter are not statistically different (*P* > 0.05) according to the *post hoc* (LSD test) comparison of ANOVA.

The *L. sakei* strains were inoculated in MRS (initial concentration ∼4 log cfu/ml) and their growth in relation to temperature and NaCl concentration was monitored through the variation of OD_600_ and modeled with the Gompertz equation (Zwietering et al., 1990). The estimates for the Gompertz parameters are reported in **Figures 2**, **3**. The figures report also the pH decrease determined by the strain growth in the different conditions with respect to the initial value of about 6.5. The presence of significant differences among the estimates of Gompertz parameters in relation to temperature and salt were tested with one-way ANOVA.

Regarding temperature (**Figure 2**), all the strains grew between 5 and 35°C but none was able to grow at 40°C. The values of standard deviation underlined a great variability in the strain performances, especially at the lower temperatures. At 5°C the coefficients of variation (CV) observed for *A*, μ_max,_ and λ were 61.4, 72.5, and 34.4%, respectively. The CV was lower for higher temperatures, but it remained rather high, especially for μ_max_ and λ. The best mean growth performances were observed for A and λ between 25 and 35°C without significant differences according to ANOVA (*P* > 0.05). On the contrary, the best performance for μ_max_ was recorded at 35°C, even if without significant differences with the value in the sample incubated at 30°C. Also, pH decrease was higher at 30°C but without significant differences with the samples at 35 and 25°C. Generally, *L. sakei* DSMZ 20017^t^ determined the lower pH decrease while the strain Chr82 induced the higher acidification, especially at 5°C.

A wide variability was observed also in relation to NaCl and the standard deviations increased with the increase of its concentration (**Figure 3**). However, all the strains were able to grow at the highest amount tested (8%) even if with a high variability in their performance, i.e., with CV of 21.5, 41.8, and 62.0% for *A*, μ_max,_ and λ, respectively. The pH decrease reflected the results observed for the parameter *A*. Also in the case of the presence of 8% NaCl, pH decreased of 1.0–1.5 units.

These variations of *L. sakei* strains in growth characteristics and acidification on MRS has already been evidenced by McLeod et al. (2008). Ammor et al. (2005) underlined the presence of relevant phenotypic differences within 36 *L. sakei* strains which regarded the growth performances and the final pH reached by the strains grown on SB medium. The same Authors revealed a noteworthy variability of the same strains in relation to the growth temperature (however, no strain was able to grow at 0 and 45°C), pH (few strains grew at 3.9) and NaCl concentration (no strain grew at NaCl 10% and about one third grew at 6.5%). By contrast, Drosinos et al. (2007) found that 7.7% of *L. sakei* strains from Greek sausages were able to grow at 45°C and in the presence of 10% of NaCl.

### Suspension of *L. sakei* Resting Cells in DM

The suspension of the cells in DM was mainly aimed to evidence the physiological differences in relation to the pre-grown conditions and to the presence or the absence of sugars. The six *L. sakei* strains were pre-grown in MRS containing glucose or ribose as fermentable carbohydrates. According to the observations of McLeod et al. (2008), *L. sakei* strains grew less in the presence of ribose, and some, among which DSMZ 20017^t^ (used also in this trial), hardly grew. For this reason, small amounts of glucose (1 mM) were added in the medium containing ribose. Under these conditions, the transcription of ribose related genes can be initiated and the pentose can be efficiently metabolized. The study of McLeod et al. (2011) demonstrated that *L. sakei* growth on ribose rather than glucose influenced not only the transcription of the genes strictly related to ribose catabolism, but also the transcription of several other genes responsible for alternative pathways was modified. In particular, many enzymes related to pyruvate metabolism were upregulated.

#### Amino Acid Metabolism

**FIGURE 4:**
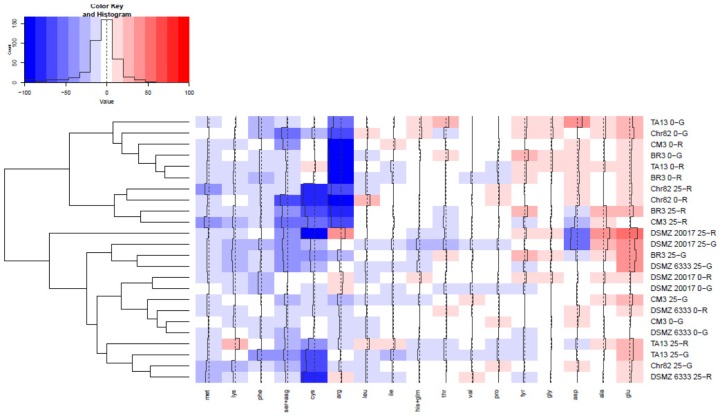
Heatmap relative to amino acid presence after 24 h of incubation. The quantitative change of each amino acid is expressed as percentage variation with respect to the initial concentration in DM (reported in **Table 1**). Increasing red intensity indicates increasing accumulation, whereas increasing blue intensity indicates increasing consumption. The dendrogram represents the hierarchical clustering process performed on the amino acid variations. A complete linkage approach on the Euclidean distance matrix was used.

The content of the different amino acids was analyzed after 24 h of incubation. The results were expressed as percentage variation with respect to the initial concentration in the DM (**Table 1**) and are reported in **Figure 4** as heat map. The results of serine and asparagine as well as histidine and glutamine are reported as sum of the percentage variation because the analytical method can not satisfactorily separate these couples of amino acids.

As it is possible to observe, many amino acids (such as glycine, proline, valine, isoleucine, and leucine) presented limited changes with respect to their initial concentration. On the other hand, serine+asparagine, arginine, cysteine together with methionine were generally the more consumed amino acids, while only glutamate and, at a lesser extent, alanine, were characterized by a constant accumulation trend. According to a cluster analysis, it was possible subdivide the considered conditions in 6 clusters.

The first grouped strains suspended in DM without sugar added, and namely BR3, TA13 (both 0-G and 0-R), Chr82 (0-G), and CM3 (0-R), which were mainly characterized by a relevant consumption of arginine. In the second cluster the consumption of arginine was accompanied by a relevant decrease of cysteine and serine+asparagine and characterized strain adapted to ribose such as Chr82 (both 0-R and 25-R), BR3, and CM3 (25-R). The strain DSMZ 20017^t^ suspended in 25-R clustered alone and was characterized by high cysteine, aspartate and serine+asparagine consumption and glutamate, arginine, and alanine accumulation. The next cluster grouped three strain in 25-G (DSMZ 20017^t^, DSMZ 6333, and BR3) which reduced the concentration of cysteine and serine+asparagine but not of arginine; moreover, these strains accumulated glutamate. The next group clustered DSMZ 20017^t^ and DSMZ 6333 suspended in DM without sugars and CM3 in DM 0-G and 25-G: in these conditions, the lower modifications of the amino acid profile of DM were observed. Finally, the last group (TA13 25-R and 25-G, Chr82 25-G and DSMZ 6333 25-R) were characterized by a relevant consumption of cysteine and by a reduction of serine+asparagine and an accumulation of glutamate.

**FIGURE 5:**
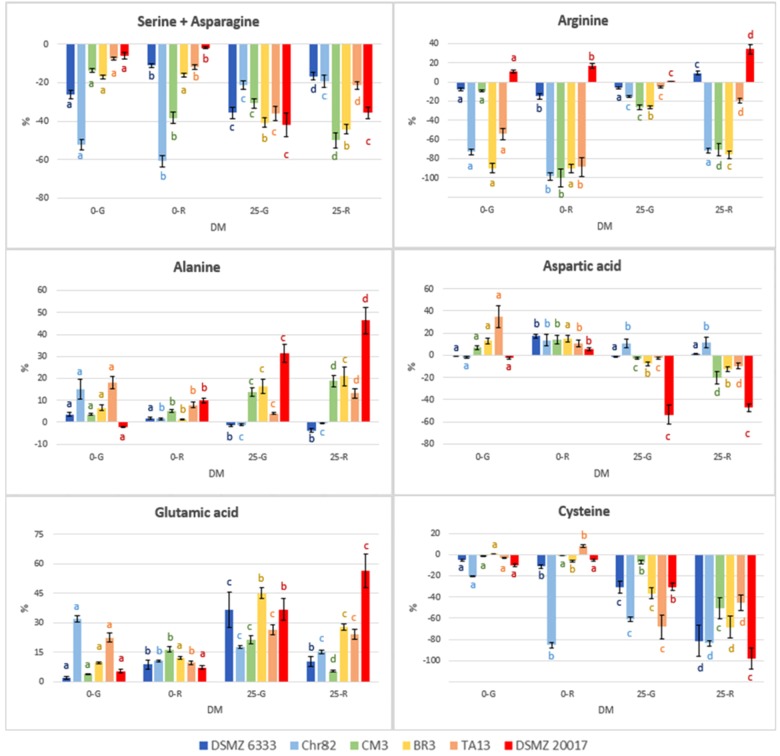
Percentage variation with respect to the initial concentration in the DM of amino acids characterized by the most relevant modification after 24 h of incubation. The standard deviations are reported. For each strain, different letters indicate significant differences in the amino acid variation with respect to DM.

**FIGURE 6:**
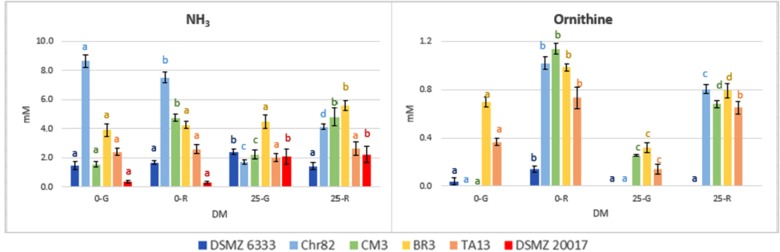
Ornithine and NH_3_ concentration in DM (expressed as mM) after 24 h of incubation. The standard deviations are reported. For each strain, different letters indicate significant differences (*P* < 0.05) according to ANOVA.

The results concerning the amino acids characterized by the most relevant modification with respect to the initial concentration are reported in **Figure 5**. Moreover, the concentration (mM) of NH_3_ and ornithine (absent in the not inoculated DM), detected with the same method used for amino acids, are reported in **Figure 6**.

The strains Chr82 and BR3 were efficient utilizers of arginine under all the condition tested. The strain TA13 preferably consumed this amino acid in the absence of sugars, while CM3 when pre-grown or incubated in the presence of ribose. The concentration of arginine was less affected by the strain DSMZ 6333, while surprisingly DSMZ 20017^t^ slightly increased its concentration. The ability to metabolize arginine through the arginine deiminase pathway (ADI), which brings to ATP production, is well known among LAB. Studies on *L. sakei* demonstrated that the species uses this pathway to improve its competitiveness and survival in meat, an environment relatively poor, for amount and diversity, in fermentable sugars but rich in arginine (Rimaux et al., 2012). Different efficiency in the utilization of arginine were also observed in two *L. sakei* strains (23K and LS25) grown in glucose limited medium; these differences were explained by the possible presence of two distinct ADI pathways, whose coexistence favored a more efficient arginine metabolism (McLeod et al., 2017). According to previous study, the ADI pathway was inhibited by low pH (Rimaux et al., 2011a; Xu et al., 2015). This trend was confirmed by the fact that higher arginine depletion was observed when sugars were not added, and, thus, when the medium was not acidified by microbial metabolisms of simple carbohydrates. In particular, the highest level of arginine consumption was observed when the strains were pre-grown on ribose. Remarkable reductions of arginine concentration were also determined by some strains (Chr82, CM3, and BR3) suspended in DM containing ribose. McLeod et al. (2011) observed in the strain *L. sakei* 23K grown on ribose an up-regulation of the genes involved in the ADI pathway, but no modification in other two strains (LS25 and MF1053).

In addition to the generation of ATP, arginine conversion can result in the equimolar production of ornithine (Rimaux et al., 2011a). In effect, ornithine, which was not added in the DM, was accumulated in the media, and especially in those inoculated with cells pre-grown on ribose, independently of the presence of the pentose in DM (**Figure 6**). The results obtained are specular to arginine being its accumulation inversely proportional to arginine consumption. The strain DMSZ 20017^t^, which did not consume arginine, did not produce ornithine.

The relation between arginine and ornithine has been confirmed in many conditions by the data reported here. Also the catabolic repression of ADI pathway by glucose (Fernández and Zúñiga, 2006) has been confirmed, given the generally lower utilization of arginine in the DM 25-G. In addition, a relevant variability in the possibility to metabolize this amino acid has been observed. One of the strain tested (DSMZ 6333) did not affect arginine content, while the strain DSMZ 20017^t^ accumulated this amino acid instead of metabolizing it, even in the absence of carbohydrates. This fact, in concomitance with the absence of ornithine, suggests that this strain lacks the ADI pathway. Ornithine was not detected in the sample Chr82 0-G (in which a high consumption of arginine was observed). Nevertheless, this was the only samples in which detectable amounts of putrescine (0.32 mM) were detected. In all the other strains was below the detection limit. Putrescine can be obtained by the direct decarboxylation of ornithine or through the agmatine deiminase pathways, whose presence have been described in *L. sakei* (Rimaux et al., 2012).

The concentration of serine+asparagine decreased in all the samples. The strains DMSZ 20017^t^, BR3 and TA13 consumed these amino acids preferentially in the presence of sugars in DM (up to 20–40%), while the higher reductions were determined by Chr82 in the absence of sugars. Aspartate, which is one of two amino acid, together with glutamate, for which *L. sakei* is prototroph, was mainly accumulated, especially in the absence of sugars. By contrast, the strain DSMZ 20017^t^ in 25-G and 25-R was responsible for a remarkable consumption of this amino acid. Cysteine decreased in DM containing sugars, especially in 25-R, while Chr82 used a great amount of this amino acid in 0-R.

The consistent decrease of some amino acids during incubation of *L. sakei* strains indicated the possible existence of other metabolic routes aimed to produce energy. Serine utilization can play a relevant role in survival during stationary phase and its catabolism can be aimed to increase the pool of pyruvate (Liu et al., 2003). The conversion of serine into pyruvate has been described in *P. pentosaceus* as the result of the activity of a serine dehydratase, without the intervention of transaminases, absent in *L. sakei* (Irmler et al., 2013). The degradation of serine with the consequent production of formate, succinate and acetate was described also in *L. plantarum* (Skeie et al., 2008). McLeod et al. (2017) observed a drastic utilization of serine and asparagine in *L. sakei* strains grown under glucose limiting conditions. Interestingly, they observed also an increase of the production of L-serine dehydratase.

Asparagine can be converted into aspartic acid with release of ammonia through the action of asparaginases, whose presence is documented among lactobacilli. Aspartate can further be catabolized through at least three pathways, two of which do not require the presence of a transaminase. Aspartate decarboxylase produces alanine and CO_2_ and aspartase produces fumarate and ammonia. Both these enzyme activities have been found in LAB (Fernández and Zúñiga, 2006). Noteworthy, alanine can be converted in pyruvate by deamination and in these trials its concentration always increased (**Figure 5**).

Also, cysteine can be metabolized to pyruvate, ammonia, and hydrogen sulfide (Fernández and Zúñiga, 2006). The enzyme responsible for this pathway (cystathionine-γ-lyase) has been found in *L. fermentum* (Smacchi and Gobbetti, 1998), *L. lactis* (Fernández et al., 2002), and *L. reuteri* (Lo et al., 2009).

The availability of pyruvate, especially in the absence of sugars or in generally limiting nutritional conditions, can be the starting point for some pathways able to provide LAB with ATP or regenerate NAD with production of ethanol, acetate, and diacetyl/acetoin (Gänzle, 2015). The generally higher activation of pathway that can supply pyruvate in the strains pre-grown or resuspended in ribose confirms the finding of McLeod et al. (2011) relative to the transcriptome of *L. sakei*. More recently, this possibility was discussed by McLeod et al. (2017) who support the hypothesis that, under glucose restriction, the pyruvate derived from amino acid metabolism can be the starting point for energy metabolic routes bringing to organic acid production. Nevertheless, in our study, a high variability in the physiological response among the strains was observed and univocal trends in relation of the sugar metabolized were not always found.

No relevant changes were observed in the concentrations of branched amino acids, whose metabolism is related to the production of aroma compounds important for the organoleptic profile of sausages. On the other hand, these metabolic pathways need the activity of amino transferases that have never been signaled in *L. sakei* (Freiding et al., 2012).

Finally, the production of ammonia was higher for Chr82 in the samples without sugar added. On the contrary, DSMZ 20017^t^ produced ammonia only in the presence of sugars (**Figure 6**). In any case, it was linearly related to the amino acid consumption (data not shown).

### Organic Acid, Diacetyl and Acetoin Production, pH and Cell Viability

The data reported in **Table 2** show the production of D- and L-lactate, acetate and formate. As expected, when glucose or ribose were supplied, they were metabolized through the homofermentative and heterofermentative pathways, respectively. Glucose is mainly converted into L-lactate while D-lactate represent about 7% of the total lactic acid in the strain Chr82, while in the other strains it was always below 4%. On the contrary, D-lactate amount is higher in the fermentation of ribose (between 6.3% in strain CM3 and 26.3% in DSMZ 20017^t^). The data reported by Malleret et al. (1998) demonstrated that *L. sakei* possesses only a L-lactate dehydrogenase (L-LDH) and a racemase able to partially convert it into D-lactate. In addition, given the specificity of the L-LDH, only L-lactate can be used for further metabolisms in nutritionally poor conditions. These findings were supported also by the work of McLeod et al. (2011). Different conclusions were observed for the strain *L. sakei* NRIC 1071 by Iino et al. (2003), who found two distinct dehydrogenases, a L-LDH and a D-LDH (the second acting later, in the stationary phase) while the presence of the racemase was excluded. The data reported here for all the six strains studied showed the presence of a high L-lactate/D-lactate ratio confirming the presence of a specific L-LDH and, eventually, of a racemase.

**Table 2:** Organic acid, diacetyl and acetoin accumulated by the six *L. sakei* strains in the defined media after 24 h of incubation. Also the pH variation and the decrease of cell viability are reported.

Strain	DM^a^	Organic acids (mM)								
		L-lactate	D-lactate	Acetate	Formate	Lactate/Acetate	Diacetyl^b^	Acetoin^b^	ΔpH^c^	Δlog cfu/ml^d^
DSMZ 6333	0-G	0.12 ± 0.08	0.14 ± 0.06	0.65 ± 0.11	0.32 ± 0.08	0.41	–^e^	–	0.14 ± 0.02	−0.65 ± 0.10
	0-R	0.10 ± 0.06	0.09 ± 0.05	0.41 ± 0.08	0.05 ± 0.04	0.48	–	–	0.19 ± 0.02	−0.74 ± 0.11
	25-G	29.99 ± 1.65	0.66 ± 0.02	3.64 ± 0.12	–^f^	13.83	9.72 ± 0.11	18.34 ± 0.54	−3.15 ± 0.05	−1.69 ± 0.09
	25-R	13.18 ± 1.15	3.12 ± 0.23	17.14 ± 0.33	–	1.02	4.97 ± 0.22	30.42 ± 1.11	−2.85 ± 0.03	−1.67 ± 0.12
Chr82	0-G	0.23 ± 0.10	0.32 ± 0.08	3.38 ± 0.14	6.53 ± 0.25	0.16	–	–	0.11 ± 0.03	−0.31 ± 0.10
	0-R	0.16 ± 0.02	0.55 ± 0.09	2.28 ± 0.07	8.11 ± 0.34	0.31	–	–	0.27 ± 0.04	−0.47 ± 0.09
	25-G	19.06 ± 0.82	2.25 ± 0.10	2.83 ± 0.26	–	11.42	11.03 ± 0.66	18.54 ± 0.81	−2.82 ± 0.06	−2.52 ± 0.15
	25-R	15.60 ± 0.23	3.26 ± 0.18	21.67 ± 0.89	–	0.92	5.0 ± 0.24	13.3 1 ± 0.81	−2.75 ± 0.08	−1.60 ± 0.21
CM3	0-G	0.23 ± 0.10	0.10 ± 0.05	0.70 ± 0.11	0.01 ± 0.01	0.47	–	–	0.21 ± 0.02	−0.62 ± 0.06
	0-R	0.08 ± 0.06	0.05 ± 0.03	1.44 ± 0.25	0.14 ± 0.02	0.09	–	–	0.31 ± 0.04	−0.18 ± 0.08
	25-G	22.18 ± 0.98	0.54 ± 0.09	1.37 ± 0.03	0.14 ± 0.04	24.73	6.37 ± 0.54	11.23 ± 1.03	−2.94 ± 0.06	−1.00 ± 0.09
	25-R	16.63 ± 0.63	1.39 ± 0.12	25.63 ± 1.00	–	0.72	16.78 ± 1.13	54.08 ± 1.22	−2.80 ± 0.09	−0.96 ± 0.11
BR3	0-G	0.10 ± 0.08	0.20 ± 0.08	0.39 ± 0.10	0.15 ± 0.03	0.76	–	–	0.20 ± 0.05	−0.02 ± 0.02)
	0-R	0.18 ± 0.03	0.22 ± 0.02	0.35 ± 0.02	0.14 ± 0.03	1.12	–	–	0.32 ± 0.04	0.09 ± 0.04
	25-G	39.69 ± 1.15	1.44 ± 0.13	2.42 ± 0.12	1.64 ± 0.09	17.00	3.9 ± 0.26	9.8 ± 0.85	−3.30 ± 0.07	−0.75 ± 0.07
	25-R	19.03 ± 0.89	2.93 ± 0.23	25.53 ± 0.66	1.02 ± 0.08	0.99	13.6 ± 1.15	64.1 ± 2.06	–3.17 ± 0.02	−0.94 ± 0.10
TA13	0-G	0.19 ± 0.09	0.10 ± 0.01	0.20 ± 0.06	0.05 ± 0.04	1.43	–	–	−0.04 ± 0.03	−1.46 ± 0.08
	0-R	0.19 ± 0.11	0.10 ± 0.04	0.36 ± 0.01	0.06 ± 0.03	0.83	–	–	0.12 ± 0.05	−1.60 ± 0.12
	25-G	36.38 ± 1.01	1.72 ± 0.07	2.55 ± 0.20	0.13 ± 0.06	19.16	4.8 ± 0.93	12.3 ± 0.18	−3.31 ± 0.09	−2.35 ± 0.21
	25-R	18.95 ± 0.66	1.78 ± 0.10	24.21 ± 0.91	0.75 ± 0.05	0.87	16.6 ± 0.73	51.6 ± 1.03	−2.99 ± 0.08	−1.96 ± 0.20
DSMZ 20017^t^	0-G	0.23 ± 0.03	0.06 ± 0.05	0.17 ± 0.06	0.25 ± 0.04	1.73	–	–	−0.11 ± 0.07	0.01 ± 0.01
	0-R	0.22 ± 0.09	0.05 ± 0.03	0.17 ± 0.01	0.07 ± 0.04	1.52	–	–	−0.10 ± 0.05	−0.10 ± 0.06
	25-G	30.14 ± 0.65	0.20 ± 0.08	2.96 ± 0.08	0.31 ± 0.02	10.25	9.3 ± 0.72	18.8 ± 0.34	−3.12 ± 0.02	−0.69 ± 0.12
	25-R	19.15 ± 0.81)	7.27 ± 0.59	25.64 ± 0.32	0.62 ± 0.04	1.04	32.7 ± 1.11	89.0 ± 1.96	−2.97 ± 0.07	−0.51 ± 0.11

*^a^Defined medium. ^b^Data are expressed as ratio between peak area of each molecule and peak area of the internal standard (4-methyl-2-pentanol). ^c^Difference between initial pH and pH after 24 h of incubation at 30°C. ^d^Difference between initial cell concentration and cell concentration after 24 h of incubation at 30°C. ^e^Not detected. ^f^Under the detection limit (0.01 mM).*

In the DM containing ribose, the presence of lactate and acetate, as expected, was almost equimolar for most strains. The strains Chr82, TA13, and CM3 presented a prevalence of acetate. This prevalence has been observed in the primary metabolism of the bacterium (McLeod et al., 2010) and explained by the increased ATP production obtained through the phosphoketolase pathway, but it can also be due to excesses of pyruvate deriving from other sources, such as amino acid metabolism (von Wright and Axelsson, 2011).

A great metabolic variability was observed in the amounts of fermentation products after 24 h among the tested strains. In the medium added with glucose, the concentration of lactate (D- and L-) varied from 21.31 (strain Chr82) to 41.13 mM (strain BR3). In the presence of glucose, also acetate was detected in amount higher than 2 mM in all the strains, except for CM3, suggesting the presence of other metabolic routes different from homolactic fermentation.

These considerations were confirmed also by the accumulation of organic acids (lactate and acetate) in the presence of 25 mM of ribose, which ranged from 33.44 mM (DSMZ 6333) and 52.06 mM (DSMZ 20017^t^).

In the absence of sugars, the amounts of lactate is irrelevant (lower than 1 mM). Different results in the same conditions were observed for acetate, which was accumulated at higher concentration than lactate and reached concentration of 3.38 and 2.28 mM in Chr82 in 0-G and 0-R, respectively. Noteworthy, the higher acetate production in these conditions corresponded with the higher formate accumulation by the same strain (6.53 mM and 8.11 mM in 0-G in 0-R, respectively). High formate concentration (more than 1 mM) were also detected in the strain BR3 in the presence of sugars. The production of formate depends on the activation of pyruvate formate lyase (PFL) pathway, favored by anaerobiosis and substrate limitation (Gänzle, 2015), which allows the production of ATP from pyruvate. The incubation of the bacteria under ordinary atmosphere, seems to be in contradiction with the activation of this route. However, recently, it has been demonstrated that PFL pathway can be functional in anaerobiosis when appropriate electron donors (ferredoxin and flavodoxin) are present (Zhang et al., 2015). Thus, it is possible that this way, when present, can be active in *L. sakei* in the presence of a molecule such as cysteine, which lowers the redox potential acting as an electron donor (Shibata and Toraya, 2015). In any case, the production of acetate (in the samples not containing ribose) and formate indicates the possibility that excesses of pyruvate deriving from amino acids can be directed into pathways able to produce energy, such as pyruvate formate liase and pyruvate oxidase metabolisms (Gänzle, 2015). The dissipation of an excess of pyruvate leads also to the production of C4 aroma compounds, such as diacetyl and acetoin (Smid and Kleerebezem, 2014). Their production in these trials was determined by SPME and the results are reported in **Table 2**. The C4 compounds were never detected in the absence of sugars. In these conditions the excesses of pyruvate deriving from amino acid metabolism were used in energy producing pathways, as demonstrated by the accumulation of lactic and especially acetic acids. When sugars were added to the medium, the exceeding pyruvate was addressed to diacetyl/acetoin production, allowing NAD regeneration (von Wright and Axelsson, 2011). The presence of acetoin was always higher (with a ratio of about 2–3:1). The strains DSMZ 6333 and Chr82 presented the lower accumulation of C4 compounds that were mainly produced in the presence of glucose. On the contrary, the remaining strains produced acetoin and diacetyl mainly in the presence of ribose. Interestingly, in the same analysis, ethanol was not detected or found only in traces (data not reported).

The decreases of pH reflected the organic acid accumulation and value between 2.94 (CM3) and 3.31 (TA13) were observed in the presence of glucose. The presence of ribose determined slightly lower decreases between 2.75 (Chr82) and 3.17 (BR3). In the absence of sugars, the pH did not significantly change.

Also, the viability of cells after 24 h of suspension in DM was monitored and the loss of viability was dramatically higher in the presence of sugars, partially attributable to the pH drop. The strains Chr82 and TA13 concentration decreased of about 2 log units and more in the presence of ribose or glucose, while the strains BR3 and CM3 were the more resistant (about one log decrease or less). In the absence of sugars added, the loss of viability was markedly lower (<1 log unit) except for the strain TA13.

## Conclusion

The metabolic heterogeneity observed among the six *L. sakei* strains confirmed the metabolic differences already observed within this species, also in relation to the genomic sequences described. All the strains could grow between 5 and 35°C (but not at 40°C) and in the presence of salt concentration up to 8%, but with growth parameters characterized by an extreme variability. In addition, the pre-grown conditions, and in particular the sugar added, induced the activation/repression of different pathways resulting in different phenotypes. The use of a DM allowed the study of amino acid metabolism. As expected, the metabolism of arginine was particularly active, even if it was not observed, under the adopted conditions, in the type strain DSMZ 20017^t^. However, other amino acids (serine+asparagine, cysteine, and methionine) were metabolized, after deamination, through potentially energetic pathways starting from pyruvate accumulation. This was demonstrated by the organic acid accumulation (especially acetate) in the DM without sugar added and also by the acetate accumulation in the presence of glucose. In addition, the presence of excesses of pyruvate deriving from amino acids lead to the accumulation of diacetyl and acetoin by all the strains when sugars were added, where the energetic needs of the cells could be satisfied by primary metabolism. These results are an important starting point to better understand the metabolisms of this species and to implement this knowledge from a productive point of view. In fact, the exploitation of the relevant phenotypic biodiversity of *L. sakei* can be a key factor for optimizing the performance of starter cultures used in meat-fermented foods.

## Author Contributions

GT carried out some of the trials of this manuscript and wrote the text. CM performed the main laboratory work and wrote the text. FB performed HPLC analysis and made figures. MM carried out some experiments. FG worked on growth curve modeling and data analysis. LG supervised the work and the manuscript writing.

## Conflict of Interest Statement

The authors declare that the research was conducted in the absence of any commercial or financial relationships that could be construed as a potential conflict of interest. The reviewer VC and handling Editor declared their shared affiliation.
